# Chemical Characterization, Antiproliferative and Antioxidant Activities of Polyunsaturated Fatty Acid-Rich Extracts from *Chlorella* sp. S14

**DOI:** 10.3390/molecules26144109

**Published:** 2021-07-06

**Authors:** Hlengiwe Vilakazi, Tosin A. Olasehinde, Ademola O. Olaniran

**Affiliations:** 1 Discipline of Microbiology, School of Life Sciences, College of Agriculture, Engineering and Science, Westville Campus, University of KwaZulu-Natal, Private Bag X54001, Durban 4000, South Africa; hhvilakazi@gmail.com (H.V.); tosinolasehinde26@yahoo.com (T.A.O.); 2 Nutrition and Toxicology Division, Food Technology Department, Federal Institute of Industrial Research, Oshodi, Lagos PMB 21023, Nigeria

**Keywords:** lung cancer, *Chlorella* sp., fatty acids, breast cancer, PUFAs, microalgae, PUFAs

## Abstract

Microalgae is a rich source of polyunsaturated fatty acid. This study was conducted to identify and isolate microalgal strain with the potentials for producing polyunsaturated fatty acids (PUFAs) and determine its cytotoxic effect on some cancer cells. The algal strain (*Chlorella* sp. S14) was cultivated using modified BG-11 media, and algal biomass obtained was used for fatty acid extraction. Gas chromatographic–mass spectrometry was used to identify and quantify the levels of the fatty acid constituents. The total content of monounsaturated fatty acids (1.12%) was low compared to polyunsaturated fatty acids (PUFAs) (52.87%). Furthermore, n-3 PUFAs accounted for (12.37%) of total PUFAs with the presence of α-linolenic acid (2.16%) and *cis*-11,14,17-eicosatrienoic acid (2.16%). The PUFA-rich extract did not exhibit a cytotoxic effect on normal cells. Treatment with the PUFA-rich extract (150 µg/mL) significantly reduced cell viability in MCF-7 (31.58%) and A549 (62.56%) cells after the 48 h treatment. Furthermore, treatment of MCF-7 with fatty acid extracts (125 and 150 µg/mL) showed a significant reduction in MDA levels, increase in catalase activities and decrease in GSH level compared to untreated cells. However, a slight decrease in MDA level was observed in A549 cells after the 48 h treatment. There are no significant changes in catalase activities and GSH level in treated A549 cells. However, a slight reduction of NO levels was observed in treated MCF-7 and A549 cells. These results indicate the potentials of PUFA-rich extracts from *Chlorella* sp. S14 to reduce viability and modulate redox status in A549 and MCF-7 cells.

## 1. Introduction

In recent years, using natural products with bioactive compounds has increased globally [1]. Intensive research has been conducted to isolate bioactive compounds from marine organisms [2,3]. Some of these organisms include fungi, bacteria, sponges, coral and algae [3,4]. Algae represent about 9% of bioactive compounds derived from marine organisms [2]. Secondary metabolites represent about 50% of drugs that have been accepted by the Food and Drug Administration [2,5]. Various novel metabolites with notable activities such as sterols, fatty acids, phenolic compounds, carotenoids and polysaccharides have been reported in microalgae [6,7,8]. These compounds have demonstrated biological activities such as anticancer [9], antimicrobial [10], antioxidant [11,12] and anti-inflammatory [11] and neuroprotective [13,14] activities.

In 2018, an estimate of 18.1 million new cancer cases and 19.6 million cancer-related deaths in the world was reported while 17 million cases was estimated for 2020 [15]. Breast cancer ranks as the most common cancer among women in the world [3,16]. In 2015, 523,000 deaths related to breast cancer was reported [17]. However, lung cancer is the leading cause of cancer-related death [16]. Chemical and radiation-based treatments are currently being used in cancer therapy [18,19]. However, a high rate of treatment failure is still a major problem [19]. Cancer incidents have continued to increase [18]; however, to address these challenges, research into safe and effective therapeutic agents are being investigated [20]. Moreover, there are indications that natural products from marine organisms may elicit anticancer activities [21].

Potential biotechnological application of microalgal lipids comprising of PUFAs has gained a lot of interest over the years [22]. Studies have revealed that microalgae exhibit anticancer properties. Custódio, et al. [23] evaluated the cytotoxicity of *Isochrysis galbana* T-ISO, *Tetraselmis* sp. and *Scenedesmus* sp. extracts. *Tetraselmis* sp. showed high n-3 PUFA content (25.90%) and the highest cytotoxic activity (IC_50_ = 58.25 μg mL^−1^) towards HepG2 cells. The antioxidant and antiproliferative activities of lipid extracts obtained from *Scenedesmus obliqus* showed a high inhibitory effect against the growth of human breast (MCF-7), hepatic (HepG2) and colon (HCT-116) cancer cell lines [22]. Sayegh, et al. [24] also investigated the anticancer and antimicrobial activity of PUFAs derived from *Nannochloropsis salina*. However, *Chlorella* strains have not been fully explored as PUFA producer neither has the fatty acids been investigated for potential antiproliferative effect. Therefore, this study sought to assess the antiproliferative and modulatory effects of fatty acid extracts from *Chlorella* sp. S14 on antioxidant enzymes in breast (MCF-7) and lung (A549) cancer cells.

## 2. Results and Discussion

### 2.1. Identification and Characterization of Microalgal Lipids

In this study, the 18sRNA gene of the isolated algal target strain was amplified and sequenced. Identification of the algal strain revealed *Chlorella* sp. S14 as the highest producer of PUFAs. The fatty acid composition of intracellular lipids synthesized by *Chlorella* sp. was identified and quantified using gas chromatography–mass spectrometry (GC–MS). The total fatty acid content is shown in Table 1. The most dominant saturated fatty acids (SFAs) were palmitic acid (20.58%), followed by myristic acid (18.55%). The high fatty acid content may be influenced by salinity, light intensity, temperature and nutrients. The study of Pandit et al. [25] revealed that 0.4 M NaCl improved the production of fatty acids in *Chlorella vulgaris* especially palmitic acid (37%) and linoleic acid (20%). High palmitic acid (33.76%) was obtained in *Botryococcus braunii* cultivated in the presence of 85 mM NaCl [26]. An increase in linoleic acid (41%) and α-linolenic acid (30%) were observed in *Scenedesmus obliquus* and *Chlamydomonas mexicana,* respectively, after supplementing growth media with 50 mM NaCl [27]. Moreover, high light intensity (750 µmol photons m^−2^ s^−1^) also triggered the production of palmitic acid (36.75%) in *Phaedodactylum tricornutum* [28].

Furthermore, the total content of monounsaturated fatty acids (MUFA) (1.12%) was lower compared to PUFA content, which represented more than 50% of total fatty acid levels (Table 1). n-3 PUFAs accounted for (12.37%) of the total PUFA content with appreciable levels of α-linolenic acid (2.16%) and *cis*-11,14,17-eicosatrienoic acid (2.16%) as shown in Table 1. Previous report has shown that light plays a significant role in the synthesis of PUFAs, which are required during the synthesis and regulation of cellular components [28]. Moreover, to maintain membrane fluidity during elevated salinity in the growth medium, microalgal fatty acid composition changes and accumulates more energy-rich compound [27]. High PUFA production observed in *Chlorella* sp. S14 may be attributed to combinatory effects of salinity (2.5 g/L NaCl) and light intensity (100 µmol photons m^−2^ s^−1^). Shekh et al. [29] reported an accumulation of PUFA (71.91%), SFA (16.66%) and MUFA (13.19%) in *Chlorella* sp. cultivated under high light intensity (100 µmol photons m^−2^ s^−1^) and low salinity (12.5 ppm). The combined effect of temperature (12 °C) and low light intensity (40 µmol photons m^−2^ s^−1^) in *Pavlova lutheri* showed higher PUFA (40.9%) levels followed by SFA (35.5%) and MUFA (19.3%) content [30].

### 2.2. Cytotoxic Effects of Fatty Acids from Chlorella sp.

**Figure 1:**
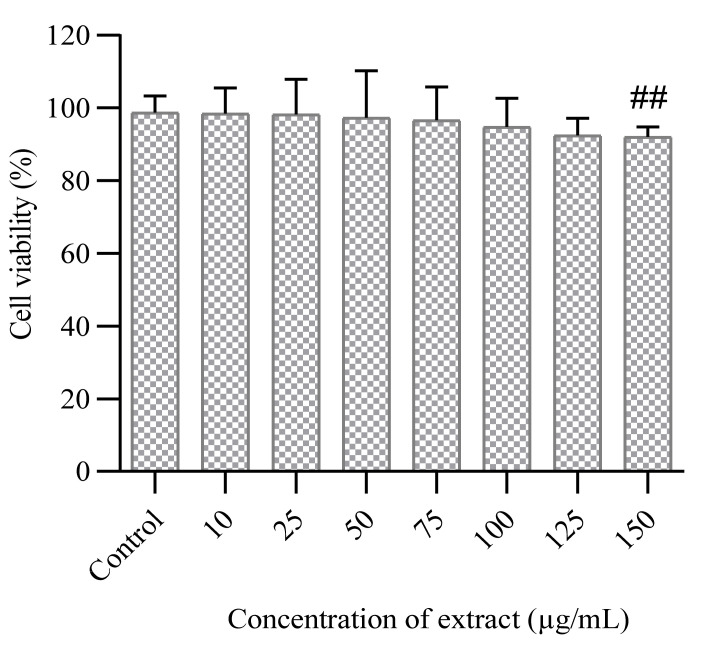
Cytotoxic effect of PUFA-rich extract from *Chlorella* sp. S14 on normal neuronal brain (HT-22) cells. Bars represent mean ± SEM. Values are statistically different at ^##^ *p* < 0.05 vs. control.

**Figure 2:**
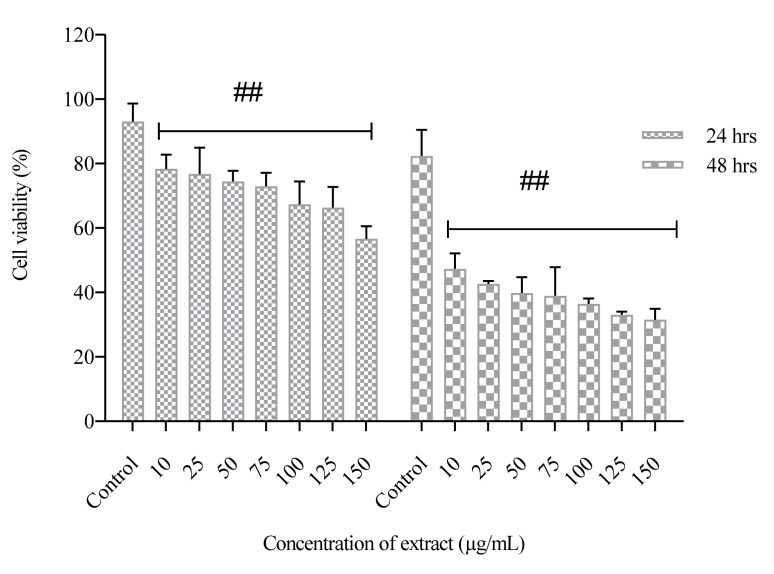
Cell viability of human breast cancer cells (MCF-7) treated with PUFA-rich extracts from *Chlorella* sp. S14. Bars represent mean ± SEM. Values are statistically different at ^##^ *p* < 0.05 vs. control.

**Figure 3:**
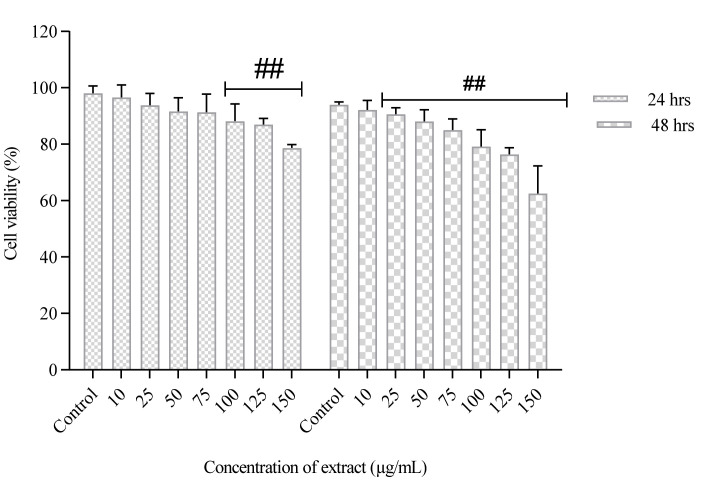
Cell viability of human lung cancer cells (A549) treated with PUFA-rich extract from *Chlorella* sp. S14. Bars represent mean ± SEM. Values are statistically different at ^##^ *p* < 0.05 vs. control.

The PUFA-rich extract did not induce cytotoxic effect on normal neuronal cells (HT-22), with cell viability of 92.1–98.7% observed in both untreated and treated cells after 48 h (Figure 1). The cell viability of MCF-7 cells was time and concentration-dependent, as shown in (Figure 2). At the highest concentration of PUFA-rich extract (150 µg/mL), a percentage cell viability of 31.5% was observed, which was lower compared to control (untreated cells) (82.3%) (Figure 2). Furthermore, the highest concentration (150 µg/mL) slightly showed antiproliferative effects against A549 cells with cell viability of 62.56% compared to the control (Figure 3).

An increase in SFA and MUFA intake has been linked with increased cancer risk and progression. However, n-6 PUFAs including linoleic acid (C18:2n-6), γ-linoleic acid (C18:3n6) and *cis*-8,11,14-eicosatrienoic (C20:3n6) have been shown to exhibit anticancer activity [31]. Lu et al. [32] also reported that linoleic acid (above 300 µM) exhibited antiproliferative effects. Hence, the antiproliferative effect of the PUFA-rich extracts used in this study against MCF-7 and A549 cancer cells may be attributed to high PUFA content. Moreover, possible synergistic effects with other fatty acids in the extracts may also contribute to the observed cytotoxic effects. Pacheco et al. reported high content of PUFA in Antarctic macroalgae inhibited the growth of MCF-7 and MDA-MB-231 cells. There are also indications that antiproliferative effects of PUFAs vary and depends on the cancer cells and concentration of fatty acid [32]. In this study, the fatty acid extract showed stronger antiproliferative effects in MCF-7 cells after 48 h of incubation than A549 cells. The action of n-3 PUFAs against cancer cells has been reported to be linked with a decrease in neoplastic transformation, suppression of growth and induction of apoptosis [33]. The study of Yin et al. [34] revealed that DHA (50 µM) reduced cell proliferation in A549 cells. Kang et al. [33] also showed that 25 µM of DHA exhibited cytotoxic effect in MCF-7 cells after 72 h of treatment. The cytotoxic effects of n-3 PUFAs against human neuroblastoma (LA-N-1) cells was linked to cell cycle arrest at the G0/G1 phase and induction of apoptosis [35]. Furthermore, α-linolenic acid reduced cell viability and changes in gene expression in four breast cancer cells (MCF-7, MDA-MB-231, BT-474 and MDA-MB-468) [36]. The observed antiproliferative effect of the PUFAs against MCF-7 and A549 cells may be linked to the combination of the PUFAs present, especially n-3 and n-6 PUFAs. Previous report has established that higher rations of n-6/n-3- PUFAs decreased cell viability of breast cancer cells [37]. In this study the ration of n-6 PUFAs was higher than n-3 PUFAs, and this may be linked to the observed decrease in survival of MCF-7 and A549 cells.

### 2.3. Effect of Crude Fatty Acid Extract on Oxidative Stress Biomarkers

**Figure 4:**
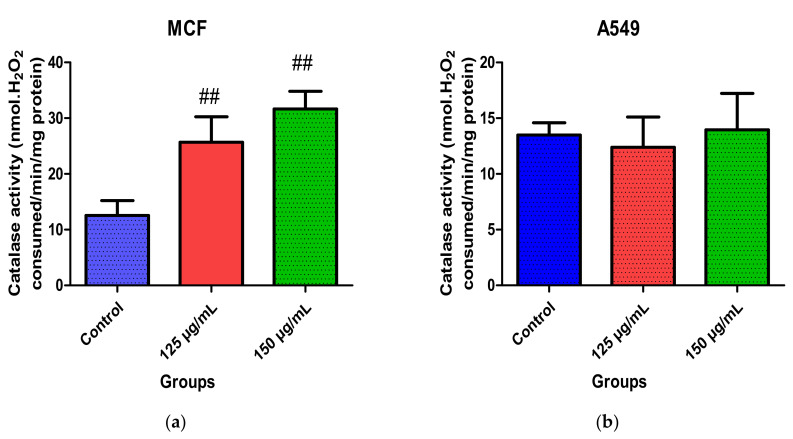
Effect of PUFA-rich extract from *Chlorella* sp. on catalase activity in (**a**) MCF-7 and (**b**) A549 cells after incubation for 48 h. Bars represent mean ± SEM. Values are statistically different at ^##^ *p* < 0.05 vs. control.

Oxidative stress has been implicated in the development and progression of cancer [38,39]. High levels of reactive oxygen species are formed in cancer cells due to an imbalance in redox status. This imbalance leads to alteration in cell metabolism, triggers malignant transformation and initiates the proliferation of cells [40]. Overproduction of reactive oxygen species and low levels of GSH, decreased catalase activities and high levels of malondialdehyde and nitric oxide have been reported to contribute to the initiation and progression of cancer [41]. In this study, a decrease in catalase activity was observed in the untreated cells (control), as shown in Figure 4a. However, treatment with fatty acids from *Chlorella* sp. S14 increased catalase activities significantly in MCF-7 cells at the concentration tested (125 and 150 µg/mL). There was no significant difference in catalase activity in A549 cells at both concentrations tested, as depicted by Figure 4b. The observed increase in catalase activity in the treated groups suggests an increase in the antioxidant defense system and response to high levels of ROS produced within the cell matrix.

**Figure 5:**
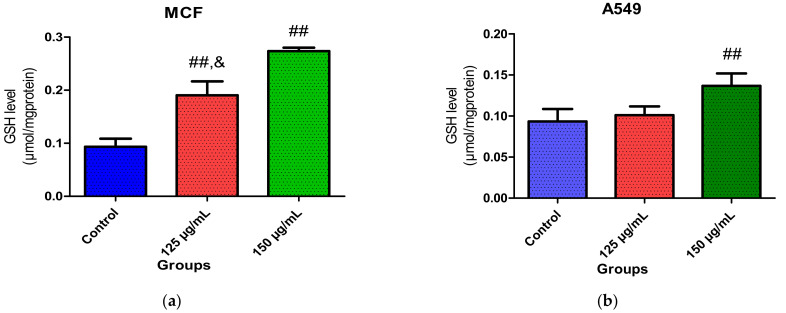
Effect of PUFA-rich extract from *Chlorella* sp. on GSH levels in (**a**) MCF-7 and (**b**) A549 cells after incubation for 48 h. Bars represent mean ± SEM. Values are statistically different at ^##^ *p* < 0.05 vs. control; ^&^ *p* < 0.05 vs. 150 µg/mL.

Similarly, a higher GSH level was observed in MCF-7 cells treated with PUFA-rich extracts, as shown in Figure 5a,b. There were no observed changes in GSH level in A549 cells after treatment with 125 µg/mL of the fatty acids (Figure 5b). However, a slight increase was observed at the highest concentration (150 µg/mL). The observed increase in GSH level may also be due to a response to defend high levels of ROS.

**Figure 6:**
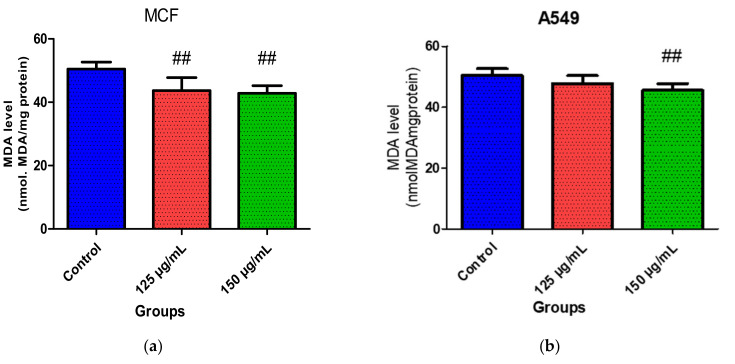
Effect of PUFA-rich extract from *Chlorella* sp. on MDA levels in (**a**) MCF-7 and (**b**) A549 cells after incubation for 48 h. Bars represent mean ± SEM. Values are statistically different at ^##^ *p* < 0.05 vs. control.

Figure 6a,b revealed MDA levels in MCF-7 and A549 cells. The production of MDA was significantly high in untreated MCF-7 cells depicted in Figure 6a. However, after treatment with the fatty acids for 48 h, MDA levels were significantly reduced, suggesting inhibition of lipid peroxidation. A slight decrease in MDA levels in A549 cells was also observed after treatment with 150 µg/mL of the fatty acids (Figure 6b). This result agrees with the report of Erukainure et al. [42], which revealed significantly low levels of MDA in MCF-7 cells after treatment with fatty acids. The observed reduction in MDA levels could be linked to high levels of PUFAs are less oxidizable compared to other fatty acids and hence may reduce the production of reactive oxygen species. Though the susceptibility of fatty acids has been linked with their degree of unsaturation, established reports have shown that the relationship between their chemical structures and degree of unsaturation is not as direct as hypothesized theoretically [43]. Furthermore, report has shown that PUFAs, especially n-3 PUFAs, show antioxidant potentials rather than being pro-oxidant; hence these fatty acids can prevent oxidative stress [44]. The report of Richard, Kefi et al. [43] also revealed that supplementation with PUFAs, including n-3 PUFAs suppressed ROS production and reduced the formation of lipid peroxidation products. In this study, the observed modulation of redox status (catalase activity, GSH and MDA levels) in treated MCF-7 and A549 cells could be linked to the presence of long-chain PUFAS in the fatty acid extracts. Furthermore, docosahexaenoic acid and arachidonic acids have been shown to inhibit ROS production [45].

### 2.4. Effect of Crude Fatty Acid Extract on Nitric Oxide Production

**Figure 7:**
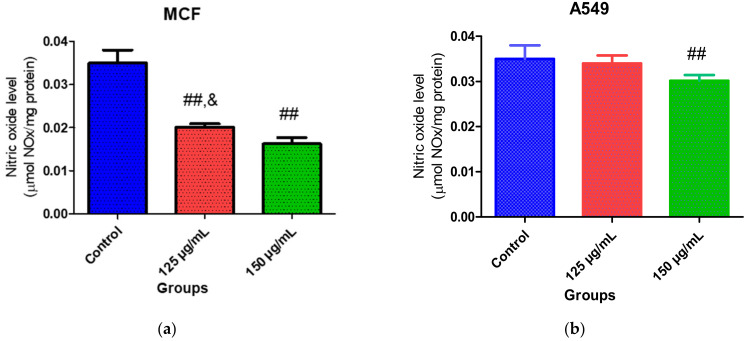
Effect of PUFA-rich extract from *Chlorella* sp. on NO levels in (**a**) MCF-7 and (**b**) A549 cells after incubation for 48 h. Bars represent mean ± SEM. Values are statistically different at ^##^ *p* < 0.05 vs. control; ^&^ *p* < 0.05 vs. 150 µg/mL.

Nitric oxide (NO) has been implicated in the etiology and progression of cancer as it induces genotoxic lesions, trigger angiogenesis and tumor cell growth [46]. Furthermore, high levels of NO contributes to nitrosative stress, which triggers DNA damage and chronic inflammation [47]. Hence, inhibition of NO production could be an important therapeutic target for the management of cancer. In this study, fatty acids obtained from *Chlorella* sp. reduced NO levels in treated MCF-7 and A549 cells compared to the untreated cells, although a slight reduction was observed in the latter (Figure 7a,b). This result agrees with the report of Ambrozova et al. [45], which revealed that PUFAs containing arachidonic acid, docosahexaenoic acid and eicosapentaenoic acid contributed to the reduction of ROS production. PUFAs, especially n-3 and n-6 PUFAs, have also been reported to inhibit NO production in macrophage and cancer cells [48,49,50]. Ohata et al. [51] also confirmed that downregulation of NO synthase expression is responsible for inhibiting NO production by n-3 PUFAs. The observed reduction of NO in both cells suggests possible prevention of inflammation, inhibition and/or reduction of nitrosative stress and protection against DNA damage.

## 3. Materials and Methods

### 3.1. Microalgae Culture Condition

Microalgal strains were obtained from culture collection in the Department of Microbiology University of Kwazulu-Natal, South Africa. The algal strains were suspended in BG-11 modified medium containing 0.5 g NaNO_3_ and 2.5 g NaCl was adjusted and standardized to an absorbance of 0.1 using a spectrophotometer (Shimadzu Corp., Kyoto, Japan) at 680 nm. A volume of 100 mL of the culture was transferred into 2000 mL Erlenmeyer flask containing 900 mL of media and was incubated for 12 days at 25 °C under fluorescent illumination provided by white light (100 µmol photon m^−2^ s^−1^) with 12 h:12 h light to dark photoperiod cycle. Biomass was harvested by centrifugation and dried using Bench Top Pro freeze-dryer with Omnitronics^TM^ (SP Scientific, Warminster, PA, USA).

### 3.2. Genomic DNA Extraction

Genomic DNA extraction was performed using Xpendition^TM^ Fungal/Bacterial DNA MiniPrep (Zymo Research, Irvine, CA, USA). Briefly, 200 µL of microalgal culture was added to ZR BashingBead^TM^ Lysis Tube (Zymo Research, Irvine, CA, USA) containing 750 µL lysis solution and vortexed for 5 min. The ZR BashingBead^TM^ Lysis Tube was then centrifuged at 10,000 rpm for 1 min. Then, 400 µL of the supernatant was transferred to a Zymo-Spin^TM^ IV Spin Filter (Zymo Research, Irvine, CA, USA) in a Collection Tube and centrifuged at 7000 rpm for 1 min. After 1 min, a volume of 1200 µL of fungal/bacterial DNA binding buffer was added to the filtrate in the collection tube. From the mixture, 800 µL was transferred to the Zymo-Spin^TM^ IIC Column in a collection tube, centrifuged at 10,000 rpm for 1 min, and the supernatant was discarded. This step was repeated twice. To prewash, 200 µL of DNA prewash buffer was added to the Zymo-Spin^TM^ IIC Column in a new collection tube and centrifuged at 10,000 rpm for 1 min. After centrifuging, 500 µL of fungal/bacterial DNA wash buffer was added to the Zymo-Spin^TM^ IIC Column and centrifuged at 10,000 rpm for 1 min. To elute DNA, Zymo-Spin^TM^ IIC Column was transferred to a 1.5 mL microcentrifuge tube and 100 µL of DNA elution buffer was directly added to the column matrix and centrifuged for 1 min at 10,000 rpm. NanoDrop 200 (UV-Vis Spectrophotometer, Thermo Scientific, Waltham, MA, USA) was then used to determine the quality and concentration of genomic DNA. 

### 3.3. PCR Amplification and Analysis of 18S rDNA Sequence

The PCR amplification was performed in T100^TM^ Thermal cycler (Bio-Rad, Hercules, CA, USA). The PCR reaction was carried out using 0.2 mM deoxynucleoside triphosphate (dNTPs), 1 mM magnesium chloride, 0.4 µm each of forward primer (5′-TGG CCT ATC TTG TTG GTC TGC-3′) and reverse primer (5′-GAA TCA ACC TGA CAA GGC AAC-3′) and 2 U of *Taq* DNA polymerase. The cycling conditions were 3 min for initial denaturation at 94 °C, followed by 35 cycles of denaturation at 94 °C for 1 min, annealing at 60 °C for 1 min, extension at 72 °C for 1 min and a final extension at 72 °C for 10 min. DNA quality and PCR products were determined by electrophoresis in 1.5% agarose gel and staining with ethidium bromide (Bio-Rad, USA) at a concentration of 1 mg/mL. The bands were visualized under ultraviolet light for the determination of the correct size of the amplified products. The PCR products were purified and sent to Inqaba Biotechnical industries, South Africa, for sequencing. The basic local alignment search tool (BLAST) was used to compare the 18S rDNA sequences to the nucleotide sequences of some known microalgae available in the GenBank database of the National Center of Biotechnology Information (NCBI). 

### 3.4. Cell Disruption and Lipid Extraction

Cell disruption and lipid extraction were carried out according to the method of Lee, et al. [52] with minor modifications. Dried biomass (1 g) was grounded using pestle and mortar, and 200 mL of solvent mixture (chloroform:methanol = 1:1 *v*/*v*) was added. The mixture was subjected to cell disruption using an Omnic sonic ruptor (Omni International the Homogenizer Company ^TM^, Kennesaw, GA, USA) at a resonance of 10 kHz for 5 min. Then 50 mL of chloroform and 25 mL of distilled water was added into the mixture to form two layers. The mixture was centrifuged for 10 min at 2000 rpm. Decantation of the mixture was done to remove the top layer while the chloroform layer was collected with a fiber filter of 0.45 µm. The extraction process was repeated for up to three times to extract more lipids. After this, the solvent was removed by evaporation in a rotatory evaporator to obtain the lipids (Dragon Lab RE 100, Beijing, China).

### 3.5. Fatty Acid Analysis Using Gas Chromatography–Mass Spectrometry

The dried lipids were subjected to esterification using 5% (*v*/*v*) sulfuric acid in methanol, with methanol: lipid ratio (30:1, *v*/*w*) and hexane as a solvent. The reaction was carried out in an orbital shaker incubator with constant stirring at 200 rpm at 60 °C for 4 h [53]. The hexane phase containing FAMEs was transferred using a pipette into a new clean Eppendorf and subjected to GC–MS analysis. The FAMEs were analyzed using gas chromatography–mass spectrometer-QP2010 SE (Shimadzu Scientific Instrument, Inc. Columbia) equipped with flame ionization detector and RXI^®^-5Sil MS capillary column. Injector temperature 250 °C, detector temperature 230 °C and nitrogen was used as a carrier gas controlled at a flow rate of 1.2 mL/min. The oven temperature was programmed at 80 °C, kept at hold for 5 min, then the temperature was increased to 290 °C and held for 5 min at a rate of 4 °C/min. The fatty acids were identified using the NIST mass spectral database and quantified by comparing the peak area with that of the external standards (C22:6 and C20:5). The relative abundance of the fatty acids was also calculated from the area percentage from the total amount of fatty acid [54,55]. Biomass from algal strain (S14) with high producing polyunsaturated fatty acids potentials was used for further studies.

### 3.6. Cell Culture

The breast cancer (MCF-7), lung cancer A549 and hippocampal neuronal (HT-22) cells were cultured in Dulbecco’s modified Eagle’s medium (DMEM), supplemented with 10% (*v*/*v*) fetal bovine serum (FBS) and 2% (*v*/*v*) penicillin. The experiments were performed with cells in the logarithmic phase of growth. The experiments were performed with cells in the logarithmic phase of growth. Cells were seeded per well in a volume of 100 μL in 96-well plates and grown at 37 °C and 5% CO_2_. Cells without any treatment were used as controls in all the experiments [3].

### 3.7. Cell Viability Assay

The cell viability was performed according to Pacheco et al. [3] using MTT (3-(4, 5-dimethylthiazol-2-yl)-2, 5 diphenyltetrazolium bromide). The extracted fatty acids were dissolved in 0.1% ethanol. Fifty microliters of fatty acids were added to 150 µL of DMEM media supplemented with 10% FBS and 2% penicillin. Normal neuronal cells (HT-22) were treated with fatty acid concentrations in 96 well plates and were incubated for 48 h at 37 °C to determine their cytotoxic effects. MCF-7 and A549 were also treated differently with fatty acid concentrations (10–150 µg/mL) and incubated for 24 h and 48 h at 37 °C. Control cells did not contain fatty acids. After incubation, the growth medium was discarded, and cells were washed with cold phosphate-buffered saline (PBS) three times, and a volume of 10 µL of MTT (1 mg/mL) and 50 µL was added in each well and incubated for 3 h. Thereafter, the growth medium was removed, and the plates were washed with PBS, after which 100 µL of dimethyl sulfoxide (DMSO) was added in each well. The absorbance was read in a microplate reader at a wavelength of 570 nm. Cell viability was determined as follows:

cell growth inhibition ratio = (1 − Abs 570treated cells/Abs 570controlcells) × 100%.(1)

### 3.8. Determination of Catalase Activity

After the treatment, cells were harvested and homogenized. Catalase activity of homogenates from treated and untreated MCF-7 and A-549 cells was determined as reported by Aebi [56]. Samples (100 μL) were added to sodium phosphate buffer (pH 7.0, 240 μL, 50 mM) and mixed with 2 M H_2_O_2_ (100 μL) that was added to the mixture. Absorbance was read at 240 nm for 3 min at 1 min interval. The decomposition of H_2_O_2_ was then monitored.

### 3.9. Determination of Reduced Glutathione (GSH) Levels

Ellman’s method [57] was used to assess the levels of GSH in untreated and treated cells. Trichloroacetic acetic acid (10%, 150 µL) was used to deproteinize the cell homogenate (150 µL). The mixture was centrifuged at 3500 rpm for 5 min at 25 °C. The supernatant (200 µL) obtained was mixed with 5,5′-dithiobis(2-nitrobenzoic acid (DTNB) in a 96 well plate. After 5 min, the absorbance of the solution was measured at 415 nm. The GSH levels of the samples were obtained from the standard curve of plotted GSH concentrations. 

### 3.10. Determination of Lipid Peroxidation

The reaction consist of a mixture containing cell homogenates (100 µL), 100 µL of sodium dodecyl sulphate (8.1%), 375 μL of 20% acetic acid, 1 mL of 0.25% thiobarbituric acid (TBA) and 425 μL of distilled water after which the mixture was incubated for 1 h at 95°C. Absorbance of the mixture was measured at 532 nm. A standard curve was generated from the absorbance of standard MDA, from which the TBARS concentration was extrapolated [58,59]. 

### 3.11. Determination of Nitric Oxide (NO) Levels

Griess reagent was used to measure levels of nitric oxide in the cell homogenates [60,61]. Griess reagent (100 µL) containing *N*-(l-naphthyl) ethylene diaminedihydrochloride (0.1%) and sulfanilamide (22%) (in 5% HCl) alongside with vanadium chloride were incubated with cell homogenates (150 µL) and was placed in the dark for 30 min at 37 °C. Absorbance was read at 548 nm and nitrite levels relative to nitric oxide concentration were measured and expressed as micromoles of nitric oxide per milligram of protein.

### 3.12. Statistical Analysis

All experiments were performed in triplicates and the results were expressed as mean ± standard deviation. The significant difference (*p* < 0.05) was tested using a one-way analysis of variance (ANOVA). All data were analyzed and plotted using Graphpad Prism 6.0 software (GraphPad Software Inc., San Diego, CA, USA).

## 4. Conclusions

The findings of this study show the antiproliferative effects of fatty acid extracts of *Chlorella* sp. via cytotoxic action against MCF-7 and A549 cells, modulation of CAT activity, GSH and MDA level, and suppression of nitric oxide production. The identification and quantification of fatty acid components of the extract revealed the presence of PUFAs, MUFAs and SFAs. The fatty acid extract contains higher levels of PUFAs than SFAs and MUFAs. Hence, this suggests that *Chlorella* sp. may be a suitable source of fatty acids rich in PUFAs with anticancer potentials. However, in further studies, the antiproliferative activity of pure and standard individual fatty component should be compared with the fatty acid extract. It is also important to investigate the biochemical mechanism of the anticancer potential of this fatty acid extract using different experimental models.

## Figures and Tables

**Table 1:** Fatty acid composition of *Chlorella* sp. S14.

Fatty Acid	Molecular Formula	Concentration (%)
Myristic acid (C14:0)	C_14_H_28_O_2_	18.55 ± 0.94
Palmitic acid (C16:0)	C_16_H_32_O_2_	20.58 ± 0.08
Stearic acid (C18:0)	C_18_H_36_O_2_	6.68 ± 0.96
Oleic acid (C18:1)	C_18_H_34_O_2_	1.12 ± 0.37
Linoleic acid (C18:2)	C_18_H_32_O_2_	17.26 ± 0.73
γ-Linoleic acid (C18:3n6)	C_18_H_30_O_2_	2.00 ± 0.11
α-Linolenic acid (C18:3n-3)	C_18_H_30_O_2_	2.16 ± 0.13
*cis*-6,9,12,15-Octadecatetraenoic (C18:3n3)	C_18_H_28_O_2_	2.09 ± 0.24
*cis*-11,14-Eicosadienoic acid (C20:2n6)	C_20_H_36_O_2_	17.36 ± 0.73
*cis*-8,11,14-Eicosatrienoic acid (C20:3n6)	C_20_H_34_O_2_	2.02 ± 0.98
*cis*-11,14,17-Eicosatrienoic acid (C20:3n3)	C_20_H_34_O_2_	2.16 ± 0.13
*cis*-8,11,14,17-Eicosatetraenoic acid (C20:3n3)	C_20_H_32_O_2_	2.00 ± 0.11
Arachidonic acid (C20:4n6)	C_20_H_32_O_2_	1.98 ± 0.00
Eicosapentaenoic acid (20:5n3)	C_20_H_30_O_2_	1.98 ± 0.00
Docosahexaenoic acid (C22:6n3)	C_22_H_32_O_2_	1.98 ± 0.00
∑n-3		12.37
∑PUFA		52.99
∑MUFA		1.12
∑SFA		45.81

Note: ∑: Sum of all n-3 or PUFA or MUFA or SFA. The fatty acid composition was determined by GC–MS analysis. Data, expressed as a percent, are means of two independent experiments ± SD.

## Data Availability

Data available on request.

## Sample Availability

Authors cannot confirm availability of sample compounds the extract is volatile cannot be kept for long hence the non-availability. Authors can confirm the algal samples.
